# Artificial Intelligence Analysis Using MRI and PET Imaging in Gliomas: A Narrative Review

**DOI:** 10.3390/cancers16020407

**Published:** 2024-01-18

**Authors:** Pierpaolo Alongi, Annachiara Arnone, Viola Vultaggio, Alessandro Fraternali, Annibale Versari, Cecilia Casali, Gaspare Arnone, Francesco DiMeco, Ignazio Gaspare Vetrano

**Affiliations:** 1 Nuclear Medicine Unit, ARNAS Ospedali Civico, Di Cristina e Benfratelli, 90127 Palermo, Italy; pierpaolo.alongi@arnascivico.it (P.A.); viola.vultaggio@arnascivico.it (V.V.); gaspare.arnone@arnascivico.it (G.A.); 2 Nuclear Medicine Unit, Azienda Unità Sanitaria Locale IRCCS, 42122 Reggio Emilia, Italy; annachiara.arnone@ausl.re.it (A.A.); alessandro.fraternali@ausl.re.it (A.F.); annibale.versari@ausl.re.it (A.V.); 3 Department of Neurosurgery, Fondazione IRCCS Istituto Neurologico Carlo Besta, 20133 Milan, Italy; cecilia.casali@istituto-besta.it (C.C.); francesco.dimeco@istituto-besta.it (F.D.); 4 Department of Oncology and Onco-Hematology, Università di Milano, 20122 Milan, Italy; 5 Department of Neurological Surgery, Johns Hopkins Medical School, Baltimore, MD 21218, USA; 6 Department of Biomedical Sciences for Health, Università di Milano, 20122 Milan, Italy

**Keywords:** artificial intelligence, gliomas, magnetic resonance imaging, positron emission tomography

## Abstract

**Simple Summary:**

The use of AI on medical images (CT, MRI, PET) has become a primary clinical and research interest. The main issues of these applications are strictly related to the reconstruction of imaging, the segmentation of tissues acquired, the selection of features, and the proper data analyses. Different approaches of AI have been proposed as the machine and deep learning, which utilize artificial neural networks inspired by neuronal architectures. Further validation of AI models for diagnosis and monitoring responses will be necessary to assess as MRI and PET/CT might provide a personalized treatment-response prediction superior to current methods.

**Abstract:**

The lack of early detection and a high rate of recurrence/progression after surgery are defined as the most common causes of a very poor prognosis of Gliomas. The developments of quantification systems with special regards to artificial intelligence (AI) on medical images (CT, MRI, PET) are under evaluation in the clinical and research context in view of several applications providing different information related to the reconstruction of imaging, the segmentation of tissues acquired, the selection of features, and the proper data analyses. Different approaches of AI have been proposed as the machine and deep learning, which utilize artificial neural networks inspired by neuronal architectures. In addition, new systems have been developed using AI techniques to offer suggestions or make decisions in medical diagnosis, emulating the judgment of radiologist experts. The potential clinical role of AI focuses on the prediction of disease progression in more aggressive forms in gliomas, differential diagnosis (pseudoprogression vs. proper progression), and the follow-up of aggressive gliomas. This narrative Review will focus on the available applications of AI in brain tumor diagnosis, mainly related to malignant gliomas, with particular attention to the postoperative application of MRI and PET imaging, considering the current state of technical approach and the evaluation after treatment (including surgery, radiotherapy/chemotherapy, and prognostic stratification).

## 1. Introduction

Gliomas are the most frequent intra-axial primary neoplasms of the central nervous system (CNS) [1]. Despite the variety of subtypes, also according to the molecular and genetic characteristics [2], some, such as the glioblastoma multiforme (GBM), have a very scarce prognosis, partially due to poor significant advancements in early diagnosis, and from a frequent occurrence of recurrence/progression after surgical and radio chemotherapy treatment [3,4]. The development of diagnostic imaging might help the management of gliomas by evaluating morphological and functional features for more accurate tumor characterization in vivo; to guide biopsy, surgery, and radiotherapy; for evaluation of disease extension; to assess relapsing disease; and for prognostic stratification.

In the past years, visual assessment represented the unique possibility of analyzing medical images to detect, characterize, and monitor diseases. Technological advances will allow visualization of the lesion with high resolution using morphological and functional imaging, also considering other derived features, to increase the possible multiparametric evaluations of gliomas significantly in a quantitative way [5,6,7]. Nowadays, the use of new quantification systems and the developments of radiomics on medical images have become crucial in research and clinical contexts. The main issues regarding the quantification workflow are strictly related to the reconstruction of imaging, the tissue segmentation, the selection of features, and the proper data analyses [8].

The advancements in radiomics analysis permit an accurate diagnostic and prognostic evaluation of the tumors, but its value in gliomas is still undetermined. Several articles have reported the potential association between tumor array and radiomic characteristics, allowing the acquisition of crucial shreds of evidence never assessed before [9,10]. Diagnosis, disease extension, vascularization, differentiation among tumor grading, and definition of tumor progression remain the main issues in neuro-oncology imaging applications. AI represents a computational model that parallels human performances on tasks, usually with automatic programming, to improve the accuracy of imaging for clinical and diagnostical management of brain tumor patients. Different classes of AI were proposed as the machine (ML) and deep learning (DL), which utilize artificial neural networks (NN) inspired by neuronal architectures [11]. Finally, new systems have been developed using AI techniques to offer suggestions or make decisions in medical diagnosis, emulating the judgment of radiologist experts [12]. It is essential to validate Artificial Intelligence (AI) on magnetic resonance imaging (MRI) and positron emission tomography (PET) techniques, which are increasingly utilized in clinical and research capacities to help define the disease and predict tumor types [13,14,15,16]. The potential advantages also rely on detecting the progression in more aggressive forms of diffuse gliomas, differentiation of pseudoprogression from actual progression, and the follow-up of aggressive gliomas [17,18].

We will focus on the available application of AI in brain tumor diagnosis, mainly related to malignant gliomas, with particular attention to the postoperative application of MRI and PET imaging, considering the current state of technical approach and the evaluation after treatment, including surgery, radiotherapy/chemotherapy, and prognostic stratification.

## 2. Materials and Methods

### Research Strategy

A comprehensive search strategy was used based on SCOPUS and PubMed databases for the indication terms: AI *OR artificial intelligence *AND glioma *OR brain tumors, along with their derivatives, with the corresponding MESH (Medical Subject Heading) terms. The search was conducted on the literature before March 2023, comprising only articles with English full text, although the research strategy did not strictly follow the criteria for a systematic review. Two authors (P.A. and I.G.V.) performed a first selection of the articles corresponding to the topic of the present review. In addition, an exhaustive study of the reference section of each article included herein was carried out. Then, all articles initially selected were checked independently by the coauthors. The reference lists of relevant papers were inspected for further studies that could fit the inclusion criteria. Using an iterative process among authors, we summarized what is known based on available case series, and retrospective and prospective studies. Considering the extreme heterogeneity and the limited amount of available information in the literature, we excluded the possibility to strictly and adequately perform a systematic review; data will be presented as a comprehensive (narrative) review.

## 3. Results

### 3.1. AI in MR Imaging

MRI represents the standard imaging for the characterization of glioblastoma, widely utilized in the diagnosis and post-treatment management of patients with glioblastoma. MRI sequences in this field include native T1-weighted (T1w) and contrast-enhanced (T1CE), T2-weighted (T2w), T2- Susceptibility Weighted Imaging (SWI), T2-fluid-attenuated inversion recovery (T2-FLAIR) sequences, and diffusion-weighted imaging (DWI), providing critical clinical information about various processes in the tumor environment. In the last decade, further MRI sequences have been developed to further characterize glioblastomas more comprehensively. These include multiparametric MRI sequences, such as dynamic susceptibility contrast (DSC), dynamic contrast enhancement (DCE), higher order diffusion techniques such as diffusion tensor imaging (DTI), and MR spectroscopy (MRS). In addition, further advantages have been reported with the availability of large field strength MRI and relative improvements in contrast-to-noise and resolution images (Table 1).

A new method of quantification of the residual tumor using multi-sequence MRI scans using automatic recording and subtraction of T1-weighted images to select the enhancing areas from inflammatory/fibrotic variations has been proposed [9]. Other studies defined a multivariate prognostic model, considering tumor remnants quantification, perfusion, radiomics, and clinical variables to improve the prognostic performance of residual tumor enhancement: the evaluation of these parameters, integrated into a predictive model to estimate the survival outcomes, was demonstrated to be an optimal method by Garcia Ruiz et al. [9].

The prediction of recurrence is one of the main issues in glioma patients, of particular interest for risk stratification and therapy management. Liu et al. identified the MRI radiomics features as potentially able to predict recurrence in glioma patients [10]. The analysis of the radiomics feature shows a value to differentiating relapsing patients from recurrence-free ones, demonstrating adequate discrimination in the training cohort and subsequent improvement in the evaluation of the validation cohort.

MR accurately assesses heterogeneous sub-regions of GBM. Ingrisch et al. presented a preoperative GBM radiomics study that analyzed the enhancing region of the tumor on T1CE [19]; other radiomics studies defined the prognostic value of multiparametric sub-regional glioblastomas [24,25,26,27]. The prognostic purpose associated all these studies by integrating radiomics with conventional clinical and genetic models with significant added value in predicting survival outcomes [28]. In this setting, ML model analysis on preoperative MRI images resulted in promising performances for predicting IDH mutation, MGMT methylation, and 1p/19q codeletion in glioma, considering the role of these key metabolic features in the differential diagnosis of brain tumors, with potential translational diagnostic and therapeutic impact [29]. ML model optimization represents a noninvasive, objective tool able to get molecular information of crucial importance for clinical management [12].

A potentially Mp-MRI-based radiomics application may be used for predicting response to chemotherapy in patients with postoperative residual gliomas and support clinical and therapy management. Zhang and coauthors selected 851 radiomics features and then applied four multivariate logistic regression models (T1 + T2 + CET1-w) to predict chemoradiotherapy response by neoplastic remnants [8].

The differential diagnosis between pseudoprogression and tumor progression is still a diagnostic issue for identifying which AI methods are well suited. Many studies in this field have successfully evaluated DWI (44,45) and dynamic susceptibility-weighted contrast enhancement measures after radiation therapy and temozolomide [30,31]. ML approaches with support vector machines (incorporating multiple measures from DWI and dynamic susceptibility-weighted contrast enhancement) similarly successfully predicted pseudoprogression [32,33]. Kim and coworkers combined structural DWI-PWI MRI, generating a radiomics model using 12 features to define pseudoprogression, obtaining an AUC of 0.85 [20]. Elshafeey et al. obtained similar results (AUC, 0.89) using a classifier of sixty radiomic features from multicentric PWI data [21].

One of the main issues for gliomas is the differentiation of infiltration margins from edema by applying conventional approaches. The ML-based definition of infiltrating tissue might guide surgical removal, biopsy procedures, and radiotherapy planning. With a voxel-wise logistic regression model, FLAIR and apparent diffusion coefficient maps appear sufficient to empower a computational model to predict recurrent disease [34]. The groups of Akbari [22] and Rathore [23] proposed a vector machine model, considering radiomics features derived from conventional and advanced MRI modalities throw a co-registration of areas of GBM recurrence to preoperative MRI. The results of such a model are some spatial maps predictive of infiltrated peritumoral tissue with a cross-validation accuracy of 90%.

### 3.2. AI in PET Imaging

The role of PET in oncology has risen substantially in the last few years. The oncology field’s most widely used radiopharmaceutical agent is F-18-labeled glucose analog 2-[18F] fluoro-2-deoxy-D-glucose (18F-FDG). This tracer has a limited clinical value in neuro-oncology for the lack of differentiation between tumor and normal brain tissue uptake. Therefore, radiolabeled amino-acids have been introduced, such as [11C]-methyl-L-methionine (11C-MET), O-(2-[18F]fluoroethyl)-L-tyrosine (18F-FET), 3,4-dihydroxy-6-[18F]-fluoro-L-phenylalanine (18F-FDOPA), or 18F-fluciclovine (18F-FACBC), to increase the diagnostic value of PET as neurooncological imaging modality [35,36,37] (Table 2).

Neural cellular absorption of these radiopharmaceuticals is associated with the operation of system L amino acid transporters (LAT1 and LAT2), governing the tissue load through distinctive metabolic pathways [41]. The concentration of LAT expression on the cellular membrane surface correlates with amino acid PET tracer absorption [41]. This mechanism is remarkably specific to neoplastic cells, and as a result is predominantly unaffected by brain-blood barrier (BBB) treatment-induced alterations and, consequently, yielding outstanding tumor-to-background contrast [41].

Numerous investigations have illustrated the additional value of amino acid PET and collaborative efforts, such as the RANO Working Group, the European Association of Neuro-Oncology (EANO), the European Association of Nuclear Medicine (EANM), and the Nuclear Medicine and Molecular Imaging Society (SNMMI), have outlined guidelines endorsing the utilization of amino acid PET for distinct diagnosis, treatment strategizing, and distinguishing tumor recurrence from treatment-associated modifications [37,42]. The RANO also put forth further suggestions for employing PET imaging to devise and oversee radiotherapy in gliomas [43].

Lohmann and coworkers evaluated the use of 18F-FET PET textural features to discriminate between pseudoprogression and true progression [18]. The authors included 34 GBM patients with suspicious tumor progression after chemoradiation (12 weeks). An ML model on four selected radiomics features from static and dynamic PET images showed 70% accuracy in the test dataset, correctly identifying all patients with pseudoprogression (AUC, 0.74; sensitivity, 100%; specificity, 40%; *p* = 0.017).

The potential use of multiparametric 18F-FET PET/MRI and AI has been assessed by Paprottka et al., describing a totally automated model, from longitudinal tumor segmentation and features extraction to classification [38]. The study included 66 patients analyzed by integrating information from 18F-FET PET, DSC-derived CBV maps of PWI MRI, and amide proton transfer-weighted (APTw) MRI imaging. An ML model was adopted for modeling data in a random forest approach. Disease progression was assessed with ROC analysis resulting in an AUC of 0.85 and an accuracy of 0.86 (sensitivity 0.91, specificity 0.70) [38]. Hotta and coauthors applied a random forest classifier, in discriminating between radionecrosis and recurrent tumors, presenting a radiomics assessment of 11C-MET PET on 44 brain lesions (gliomas and metastases) [39]. Utilizing this approach, the radiomics demonstrated markedly superior sensitivity, specificity, and accuracy (90.1%, 93.9%, and 92.2%, respectively) compared to conventional tumor/background ratio (TBR) assessment, which yielded a sensitivity of 60.6%, specificity of 72.7%, and accuracy of 63.6% (with a designated cut-off value of 2.83).

More recently, textural features extracted from postoperative 18F-FDG PET, 11C-MET PET, and MRI scans were also evaluated using radiomics-based models by Wang and coauthors [44] in 160 glioma patients, to test the performance of discrimination between tumor recurrence and radionecrosis. The integration of clinical and derived imaging information was proposed using a logistic regression model. Finally, the age, TBR mean of 18-FDG PET, TBRmax of 11C-MET PET, and other twelve textual features were significant contributors to the discrimination of tumor recurrence and RN (*p* < 0.001) both in primary and validation cohorts.

Russo et al. demonstrated the feasibility of a new statistical methodology for selecting significant descriptors, using a solid statistical classifier (discriminant analysis-DA), to propose a model of prediction of tumor grading (low versus high grade) on 11C-MET PET studies [17]. Although the study was performed for diagnosis assessment, the application of this model might be translated into the post-treatment evaluation. The innovation of this study is the use of discriminant analysis [45] as an ML set of rules adopting a k-fold strategy and comparing two different segmentation algorithms for tumor identification. The final aim was to avoid intra- and inter-user variability that may occur with manual delineation: (i) a VOI obtained using an automatic thresholding method, and (ii) a fixed ROI of 81 voxels centered on the SUVmax voxel to eradicate the dependency on the volume. The highest performance was obtained using the VOI, demonstrating no particular advantages via a homogeneous fixed ROI rather than a segmentation algorithm that obtains different volumes among patients’ studies.

11C-MET PET was used to detect recurrent brain tumors and differentiate them from radiation necrosis by a radiomics approach. Hotta et al. extracted forty-two PET features of forty-four brain lesions using a random forest classifier and the diagnostic performance evaluation with a 10-fold cross-validation scheme [39]. Radiomics and T/N (tumor/noise) ratio evaluation showed sensitivities of 90.1% and 60.6% and specificities of 93.9% and 72.7%, with areas under the curve of 0.98 and 0.73, respectively. Gray level co-occurrence matrix dissimilarity was the most pertinent feature for diagnosis. 11CMET PET radiomics generated an outstanding outcome for discerning relapsing tumors from radiation necrosis, which outperformed the T/N ratio assessment.

To develop an integrated model for discriminating recurrence from radionecrosis, Wang et al. highlighted integrated 18F-FDG PET, 11C-MET PET, and MRI images [44]. The study defined 15 features significantly associated with tumor recurrence. This combined model considered the radiomics signature, the mean TBR of 18F-FDG, the maximum TBR of 11C-MET PET, and patient age, demonstrating practical insight of recurrences (AUC of 0.988, 95% CI of 0.975–1.000). Application in the validation cohort showed good differentiation (AUC of 0.914 and 95% CI of 0.881–0.945). Decision curve analysis showed that this integrated model was clinically valuable.

Some applications of ML on PET imaging were also performed for the study of attenuation correction (AC) and low-count image reconstruction [40]. The research development in this field regards generating synthetic CT from MR or non-AC PET for PET AC and direct conversion from non-AC PET to AC PET [40,46]. Despite the recent advances of integration of AI in the new workstation and systems commercially available, the clinical impact of these AI methods has to be confirmed by many datasets and prospective studies, considering all potential bias that may occur in these analyses and the reproducibility and real added value on clinical outcome.

## 4. Discussion

Despite the rapid diffusion of AI processes, serious technical difficulties in using these sophisticated algorithms in clinical practice have been reported in the literature. Firstly, the software must be combined with the radiologist’s workflow features. Additionally, a lot of segmentation and radiomic models need much processing time, manual intervention, and a variety of in-house pipelines. Due to the absence of specific guidelines on image segmentation, the current pioneering status induced the researchers to develop some methods with good performance, for example, wavelet analysis and transformation, neural networks, or genetic algorithms [47]. After surgery, MRI with paramagnetic contrast agents is the standard method to evaluate neoplastic growth and therapy response [48]. Classically, the Response Assessment for Neuro-Oncology (RANO) and Macdonald criteria for GBM assessment take into consideration the product of the two maximum diameters of the enhancing tissues [49]. The heterogeneity of glioma pathogenesis and its aggressiveness with lack of therapy responses in most patients raises the need to classify effective treatments using the available surveillance tools accurately. Compared to linear methods, volumetric MRI has demonstrated good performance in determining the neoplasm dimensions. Furthermore, the volumetric analysis of tumor was recognized as the best predictor of outcome than linear-based techniques, as demonstrated by Dempsey et al. [50]. In addition, another study that proposed a semi-automatic approach to assess the brain tumor size was able to reduce inter-observer variability [51], demonstrating the added value on the delineation of size and quantification of the tumor [52]. The last years have assisted a widespread application of radiomics in prognostic stratification of GBMs before therapy, given the capability of this method to permit quantifiable analysis of radiological images, then converting this information into a large number of standardized features [53]. In clinical practice, the extent of resection should be evaluated with an early postoperative MRI scan and also PET scan, as complementary imaging, to improve the differentiation between inflammatory reparative changes and residual tumors. Quantitative evaluation of the residual tumor to obtain more continuous variables than dichotomous values (qualitative assessment) may help identify the best therapy and prognosis approach, considering the potential reproducibility and comparability in multicenter studies. Therefore, identifying additional prognostic values using advanced image analyses of the tumor remnants at the postoperative MRI and PET is becoming essential.

ML foresees the mining of quantitative features with different degrees of complexity based on the adopted statistical approach applied to images, such as histogram- and texture-based features, fitted biophysical models, spatial patterns, and DL features, to predict information on the tumor-infiltrating boundaries, molecular markers, and also prognosis, of vital interest for patients’ management. On the other hand, neuro-oncological radiomics DL methods generally require less domain-specific data than the explicitly engineered features for traditional ML, thus permitting them to make predictions without explicit feature selection or reduction steps [54].

Empowering these methods based on AI permits computers to make decisions automatically. From a translational point of view, DL methods mirroring the human visual cortex neural networks, applied to neuroimaging, have been related to an improvement in neurosurgical management [55,56]. Some potential applications proposed are associated with diagnosis, tumor grading and staging, outcome predictors, and (even if still under evaluation in radiogenomics) associating genetics with imaging features [12].

Many ML models are developed in supervised forms, consisting of algorithms trained on different diagnostic and prognostic applications (e.g., survival estimation, grading, tumor enhancement, or necrosis). These models need adequate samples of the various applications to “learn” and categorize different data. Supervised ML methods include logistic regression, support vector machines, random forests, and others software potentially useful in clinical settings [57].

Generally, the traditional supervised approaches are applied after feature reduction to minimize model complexity and avoid overfitting (i.e., memorizing the training sample cases rather than learning the relevant pattern). Despite the power of these approaches, extensive, domain-specific, expert knowledge about the underlying biologic basis of the process is necessary. Some studies focused on unsupervised ML algorithms as k-means clustering, which can provide new groups of categories from complex data sets [54].

The development of computer systems in terms of electronic power, graphical processor, and mathematical optimization methods provided new advancements in neural network models to contain many intermediate layers, thus differentiating DL from outmoded neural networks. The introduction of iterative processes permits the improvement of model weights (“back-propagation”) to accurately recognize low/intermediate level image information, determining a maximization of the classification performance. In the case of image-based problems, a subclass of feed-forward neural networks, namely the feed convolutional (CNNs), have been used. Compared to the traditional ML approaches, DL models can dash and do not require much manual intervention, but a large quantity of labeled training data is necessary [54].

Potentially able to discriminate patterns by incorporating relevant multimodal imaging with clinical/pathology/molecular information that humans cannot assess, AI methods represent a promising tool for the future of radiology in the precision medicine era. AI’s objective in brain tumors focuses on the potential advantage of generating a patient-tailored prediction about molecular markers (precision diagnostics), prognosis, and specific treatment recommendations (precision therapeutics). Using a fully automated system might also help the treatment monitoring and therapy management with more precise quantitative reporting tools to promptly track changes in conventional and advanced imaging parameters and patterns derived, for example, from DL. To give substantial advantages to patients’ clinical and diagnostic evaluation, a complete synergy between humans and computers will be necessary to interpret information correctly from images, AI tools, and health records [54]. The first objective of this research line is to improve clinical outcomes for patients affected by brain tumors through progress in diagnostic, monitoring, and therapy management. AI methods merging medical, radiomic, and genomic information into predictive models might have an impact on monitoring tailored therapies. However, many challenges still exist, and further efforts are necessary for a translational application.

Despite the potential ability of AI diagnostic resources in the clinical and diagnostic setting, these systems still require substantial human oversight and supervision because they cannot always understand the clinical context and related clinical questions.

Additionally, as data specialists, radiologists must provide continual, real-time feedback to update the AI system [58], serving as part of the “checks and balances” between humans and machines. Early prototypes of generalized AI have been proposed in the literature [59]. While AI is still far from generalized effectiveness, it holds promise to one day improve neuroimaging at all levels, from processing and diagnosis to education and management [11]. Further validation of AI models for monitoring responses will be necessary to assess as MRI and PET/CT might provide a personalized treatment-response prediction superior to current methods.

## 5. Conclusions

Future AI applications will focus on developing systems able to perform imaging quantification and incorporate the multi-omics information deriving from radiology, nuclear medicine, electrophysiology, laboratory findings, and clinical data and eventually create a comprehensive clinical-radiology report, and support the clinical decisions in multi-disciplinary discussions, integrating different AI systems trained on a vast wealth of neurological settings. Future physicians will have to be trained in the appropriate use of AI to integrate its use into daily practice during the coming years.

## Figures and Tables

**Table 1:** AI application for MRI-based imaging in gliomas.

Authors	Imaging Technique	Clinical Setting	AI Methods	Main Findings	Sample
Garcia Ruiz et al. [9]	MR	Prognosis stratification	The 3D distance transform of the volume of interest (VOI). Radiomics extraction was performed with Pyradiomics v2.1.2 for Python	The prognostic value of several imaging and clinical data was studied both individually and combined to estimate the survival outcomes, demonstrating that the residual enhancement thickness and radiomics signatures complemented clinical data for prognosis stratification.	144 GBM
Liu et al. [10]	MR	Prediction of recurrence	(LASSO) regression model for data dimension reduction, feature selection, and radiomics feature analysis—MIM system and MatLab	A prediction radiomics model that may guide the therapy management was assessed, leading to the identification of features that potentially could help in discriminating recurrence from recurrence-free.	129 patients
Ingrish et al. [19]	MR	Prognosis stratification	Tumor segmentation using the Medical Image Interaction Toolkit. Automated feature extraction pipeline with Python.	Baseline contrast-enhanced T1-weighted MR includes hidden prognostic information, which can be used to build prognostic models by radiomic analysis with random survival forests.	66 GBM
Zhang et al. [8]	MR	Prediction of treatment response	Image normalization and segmentation with 3D-Slicer 4.10.2 platform. Radiomics model developed with R-4.0.3	Radiomics models applied to preoperative multiparametric MR images have a potential role in predicting the response to concurrent radiotherapy and chemotherapy in patients with residual glioma. These models may support the personalization of treatments, especially to help patients initially predicted to be treatment-insensitive avoid the toxicity of chemoradiotherapy.	84 patients
Kim et al. [20]	MR (DWI-PWI)	Differentiation between pseudoprogression and true progression	Texture analysis (162 features). selected after training and external validation sets	Incorporating DWI and PWI images into a radiomics model improved diagnostic performance to differentiate pseudoprogression from early tumor progression.	61 GBM
Elshafeey et al. [21]	MR (PWI)	Differentiation between pseudoprogression and true progression	Support Vector Machines with linear kernel and C5.0 models were constructed using the features selected by the MRMR analysis	Radiomics information extracted from PWI images could be used to build a clinically-relevant predictive model to discriminate pseudoprogression from true progression.	98 GBM
Akbari et al. [22]	MR multiparametric	Prediction of recurrence	GLISTR software image analysis technique incorporating probabilistic imaging and biophysical models	A multidimensional machine model adopting co-registration of areas of GBM recurrence to preoperative MR was proposed, determining predictions of early recurrence with sensitivity 91% and specificity 93%.	31 GBM
Rathore et al. [23]	MR multiparametric	Prediction of recurrence	GLISTR software image analysis. Multidimensional pattern classifier trained on features of the voxels of N-ROI and F-ROI using support vector machines	This study presents a model for estimating peritumoral edema infiltration using radiomics signatures, reaching about 90% accuracy.	31 GBM

**Table 2:** AI in PET imaging of Gliomas.

Authors	Imaging Technique	Clinical Setting	AI Methods	Main Findings	Sample
Lohmann et al. [18]	FET PET	Differentiation between pseudoprogression and true progression	Radiomics feature extraction with Pyradiomics. Validation using 5-fold cross-validation and Machine Learning model Tree-based Pipeline Optimization Tool (TPOT)	The radiomics model correctly diagnosed all patients with pseudoprogression in an independent test cohort without the need for dynamic FET PET scans.	34 GBM
Paprottka et al. [38]	FET PET/MRI	Differentiation between pseudoprogression and true progression	SRI24 atlas space and resampled using a rigid, mutual information-driven registration with the open-source ANTs software. BraTS Toolkit and subsequent Random Forest classifier	ML model combining data from FET PET and advanced MRI imaging techniques in a random forest approach assessed the disease progression with sensitivity 91% and specificity 70%.	66 patients
Hotta et al. [39]	MET PET	Differential diagnosis between radionecrosis and recurrent tumors	Image analysis using the LIFEx package. Machine learning with Random Forest classifier—10-fold cross validation	MET PET radiomics signatures outperformed T/N ratio evaluation: sensitivities of 90.1% and 60.6%, and specificities of 93.9% and 72.7%, respectively.	44 brain lesions (gliomas and metastases)
Wang et al. [40]	FDG PET, MET PET and MRI	Differential diagnosis between radionecrosis and recurrent tumors	In-house texture analysis software, called AnalysisKit. least absolute shrinkage and selection operator (LASSO) method for features selection and 10-fold cross validation. glmnet” package on R-Studio Software	A logistic regression model combining clinical (patient age) and derived imaging information (the radiomics signatures, the TBRmean of FDG PET and the TBR maximum of MET PET) provided a good discrimination between radionecrosis and recurrent tumors with an AUC of 0.988.	160 gliomas
Russo et al. [17]	MET PET	Diagnosis assessment (tumor grading)	LIFEX for segmentation. A mixed descriptive-inferential sequential approach for feature selection and subsequent machine learning model based on discriminant analysis	An ML model based on discriminant analysis was proposed with the aim of reducing intra- and inter- user variability: the best result was related to a VOI obtained using an automatic thresholding method.	66 patients
